# A Virtual Reality Food Court to Study Meal Choices in Youth: Design and Assessment of Usability

**DOI:** 10.2196/12456

**Published:** 2019-01-09

**Authors:** Margaret Allman-Farinelli, Kiran Ijaz, Helen Tran, Hermes Pallotta, Sidney Ramos, Junya Liu, Lyndal Wellard-Cole, Rafael A Calvo

**Affiliations:** 1 Charles Perkins Centre School of Life and Environmental Sciences University of Sydney University of Sydney Australia; 2 School of Electrical and Information Engineering University of Sydney Sydney Australia

**Keywords:** virtual reality, nutrition promotion, food policy, take-out food, obesity, young adults

## Abstract

**Background:**

Regular consumption of take-out and fast foods with sugary drinks is associated with poor quality diets and higher prevalence of obesity. Among the settings where such food is consumed is the food court typically found in shopping malls prominent in many countries.

**Objective:**

The objective of this research was to develop a virtual reality food court that could be used to test food environmental interventions, such as taxation, and ultimately to facilitate the selection of healthier food choices.

**Methods:**

Fourteen food courts in Sydney, Australia were selected to include those in the city center and suburbs of high and low socioeconomic status. Researchers visited the courts to collect information on number and type of food outlets, all menu items for sale, cost of foods and beverages and sales promotions. This information was used to assemble 14 food outlets typically found in food courts, and representative menus were compiled. The UNITY gaming platform was used to design a virtual reality food court that could be used with HTC VIVE goggles. Participants navigated the virtual reality food court using the head-mounted display, keyboard, and mouse and selected a lunch meal, including food and beverage. A validated questionnaire on presence within the virtual reality food court and system usability was completed at the end of the session. The constructs for presence included a sense of control, sensory fidelity, realism, distraction, and involvement. Questions were rated on a scale from 1 (worst) through 7 (best) for each of 28 questions giving a maximum total score of 196. The systems usability scale (SUS) that gives a final score out of 100 was also assessed.

**Results:**

One hundred and sixty-two participants with a mean age of 22.5 (SD 3.1) years completed the survey. The mean score for total presence was 144 (SE 1.4) consisting of control: 62.1 (SE 0.8), realism: 17.5 (SE 0.2), involvement: 9.6 (SE 0.2), sensory fidelity: 34.9 (SE 0.4), and distraction: 24.0 (SE 0.3). The mean SUS was 69 (SE 1.1).

**Conclusions:**

Virtual reality shows promise as a tool to study food choice for test interventions to inform practice and policy.

## Introduction

The world is currently experiencing an obesity epidemic [1]. An association between transactions for fast food meals per capita and population body mass index has been demonstrated in the member countries of the Organization for Economic Cooperation and Development (OECD) [2]. Policies that aim to limit the consumption of these foods are recommended. Among the suggested approaches are energy and nutrition labeling, regulation of food advertising, incentives for healthier choices, taxation, and reformulation [3].

Adolescents and young adults show the highest rates of weight gain [4], have the poorest quality diets [5] and are more likely to eat meals out including take-out meals [6]. They are an age group vulnerable to advertising [7] and they are sensitive to price, [8] so that cheap and tasty meals hold considerable appeal. Clearly, among the actions to be taken to curb obesity rates in this demographic must be intervention in the fast and take-out foods sector. One venue where young people congregate in many OECD countries is the shopping mall and its food court. Conducting experiments in this setting is met with many barriers and food outlet owners may require evidence that any measures imposed on them will achieve the intended aim of changing rates of overweight and obesity. A number of countries have enforced the display of calorie counts on fast-food menu boards [9]. While these have led to greater awareness of energy contents and better choices by those who use them, only about 30% of people do so [10]. We propose that virtual reality may offer a means to test the potential efficacy of different policy approaches before they are implemented in real-world trials.

Previous research with virtual reality supermarkets in the Netherlands, New Zealand, and United Kingdom (UK) and a virtual reality buffet in the United States has shown high acceptability of such a platform and report that behavior is similar to that in the real world [11-15].

The aim of this study was to develop a virtual reality food court (VRFC) and to test its usability, and factors associated with presence in a sample of young adults. Presence in virtual reality is the phenomenon of being present in the computer-generated environment rather than the real world around oneself [16]. Establishing presence was believed to be an important step if the virtual food court environment will be used to predict food choices in the real world.

## Methods

### Development of the Virtual Reality Food Court

Fourteen food courts that included those in the central business district and across the suburbs of a major global city with a population of five million were visited. Food courts in suburbs of both higher and lower socioeconomic status were selected. Information was collected on the number and type of food outlets, all menu items for sale and cost of foods and beverages. Photographs of all displays were taken. The nutritional composition of foods and beverages for sale was compiled into a database. Nutrition information was obtained from both the Australian Food, Supplement and Nutrient Database (AUSNUT) 2011-13 [17] and the commercial outlets’ websites. This included both macronutrients and micronutrients of interest because of potential deleterious effects (ie, energy, protein, total fat, saturated fat, carbohydrate, total sugars, dietary fiber, and sodium). The database values included a nutrient composition for the serving sizes for sale as well as the composition per 100g.

**Figure 1:**
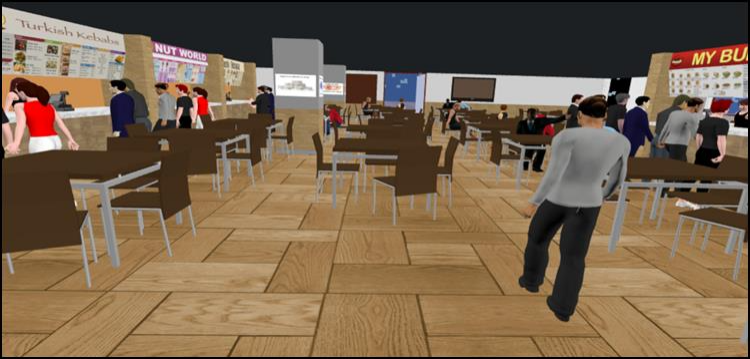
Overview of the virtual reality food court.

**Figure 2:**
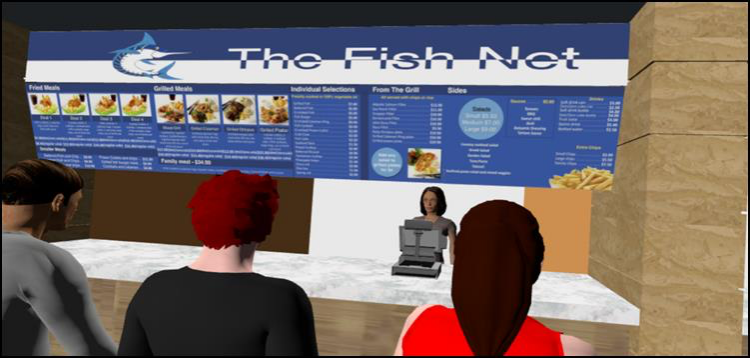
An individual food outlet within the virtual reality food court.

The similarities in food outlets across all food courts allowed compilation of menu items into 14 representative stores. As an example, there were typically three major chains of stores selling chicken fast-food products and a menu incorporating food items for all three was used for one store. This meant it sold chicken burgers, chicken wraps, and rolls, chicken salads, whole chicken, portions of chicken and nuggets that were fried, roasted and grilled as well as side dishes such as vegetables, salads, and sauces. The names selected for the stores were purposely different to any commercial names so as not to infringe any registered trademarks. These were a burger outlet (My Burger), a fried and barbecued chicken outlet (Clucky Fried Chicken), a sandwich chain outlet (Sandwich King), an independent sandwich outlet (Sandwich House), doughnut outlet (Donut World), muffin outlet (Muffin Mania), café outlet (Glory Coffee), salad bar outlet (Salad Soul), juice bar outlet (The Juice Team), seafood outlet (The Fish Net), sushi outlet (Sushi Besuto), an outlet selling Asian cuisine (Little Asia), a kebab outlet (Turkish Kebabs) and an outlet selling Indian cuisine (Taste of India). The menu boards were made to resemble those in the real-world food court using the photo images collected as a reference point. The pricing of food items was based on the prices collected during the initial visits to the real food courts and confirmed on the outlet's websites. Screenshots of the overview of the VRFC and an individual outlet with menu boards are shown in Figures 1 and 2. In total 515 foods and condiments and 219 beverages were available for sale across the 14 food outlets.

The gaming development platform, UNITY 5.4.0, was used to construct the VRFC which was made compatible with an HTC VIVE head mounted display (HMD). The Asus G751JY was used to host the VFRC. The VRFC was developed to allow various menu boards and promotional posters to be uploaded so that modified versions of the same outlets with the same menus can be used in randomized controlled trials of the court under varied conditions. For example, boards that have taxed conditions on sugar-sweetened beverages can be loaded; posters that show the public health dangers of excess sugars can be displayed within the court.

### Study Procedure

Once developed the food court was tested in a sample of young adults recruited in the real food court of a large urban university (>60,000 students). The venue was selected as it provided the sounds and smells that one encounters in a real food court. Researchers distributed recruitment flyers to participants at the food court during typical lunch hours of 11 am until 2 pm. To be included in the study, subjects had to be aged 18 to 35 years of age. Those who regularly experienced motion sickness were excluded. All subjects gave informed consent. The study was approved by the Institutional Human Research Ethics Committee (Project 227).

Subjects received instructions regarding the HTC VIVE HMD and navigation in the virtual reality food court. They were asked to select a lunch meal, including food and beverage, up to the value of Aus $10 that was adequate to cover the costs of a sandwich, burger or hot meal choice and beverage or a meal deal with beverage included After they had made their choice the participants were asked to complete questionnaires regarding the system usability and presence in the food court. Participants received Aus $10 as compensation for their time.

### Study Measures

#### Presence and System Usability

Each subject had their age, gender, and meal purchases (ie, food and beverage) recorded. All participants completed an online questionnaire that included questions on usability and presence. The presence questionnaire was based on the 32 item Presence Questionnaire developed by Witmer and Singer [16] which has been demonstrated to have high internal validity and repeatability. The factors contributing to presence are control in the activities of the virtual experience, realism of the environment, the sensory fidelity of the environment and distraction in the virtual environment. Items 6,15,16 and 17 of the questionnaire were excluded in this study because no sound and touch were offered by the virtual reality game with sound and food smell restricted to those within the chosen real food court where the experiments were conducted. All 28 questions were scored on a seven-point scale.

Nine items on system usability from the scale originally developed by Brooke [18] (question 2 to 10) were included and participants rated questions on a scale with strongly disagree at one end and strongly agree at the extreme end. The item concerning frequent use of the court was excluded as it was not expected this virtual reality experience would be used in the same participants on repeated occasions.

#### Statistical Analysis

For each of the questionnaire items measuring presence, the response for each score of 1 to 7 was calculated and tabulated. These were then grouped and analyzed according to the factor it measured: control (items 1, 2, 3, 7, 12-14, 21, 25-27, 29, 31), realism (11, 12, 14, 22), sensory fidelity (4, 5, 10, 14, 18-20), and distraction factors (8, 9, 24, 28-30). A subscale item of involvement was also scored (23, 32). For responses to items 8, 9, 11, 22, 24, 25, 28, and 29, a higher score is actually a negative outcome for the VRFC. Thus, to calculate the total score and mean for each factor the scales have been reversed so that score 7 would now correspond to score 1 and score 1 would now be score 7. Usability was assigned an overall score out of 100 in accordance with the scoring method. The number of foods and beverages purchased from each outlet were compiled. All statistics were conducted using Microsoft Excel.

## Results

### Participant Characteristics

A total of 162 young adults gave consent to participate in the study and completed the presence and the usability questionnaire and all of these results were included. However, only 157 (96.9%) completed their purchase of food. Failure to complete was due to feeling uncomfortable wearing the HTC VIVE HMD and nausea.

### Food and Beverage Purchases

All 14 food outlets were visited for lunch. Flavor of India was the most popular outlet for food and beverages. The fish burger (5.1%) from The Fish Net was the most common food item purchased. Canned regular cola was the most commonly purchased drink (19.1%) followed by all types of fruit juices (15.3%). Table 1 shows the number of purchases from each outlet.

### Presence

Table 2 shows the results for each of the presence questions. Table 3 shows the mean total presence and the scores for the factors (ie, control, sensory fidelity, realism, distraction, and involvement).

### Usability

The second section of the VRFC questionnaire measured system usability and the mean systems usability scale score was 69.0 (SE 1.1) out of a possible 100.

**Table 1:** The frequency of food and beverages choices in the virtual reality food court.

Outlets	Food, n (%)	Beverage, n (%)
Clucky Fried Chicken	20 (12.7)	8 (5.1)
Donut World	2 (1.3)	5 (3.2)
Flavor of India	30 (19.1)	35 (22.3)
Glory Coffees	1 (0.6)	17 (10.8)
Juice Team	1 (0.6)	3 (1.9)
Little Asia	8 (5.1)	9 (5.7)
Muffin Mania	9 (5.7)	13 (8.3)
My Burger	16 (10.2)	9 (5.7)
Salad Soul	5 (3.2)	5 (3.2)
Sandwich House	1 (0.6)	3 (1.9)
Sandwich King	11 (7.0)	8 (5.1)
Sushi Besuto	18 (11.5)	13 (8.3)
The Fish Net	22 (14.0)	21 (13.4)
Turkish Kebabs	13 (8.3)	8 (5.1)

**Table 2:** Presence questionnaire items with the percentage of respondents for each of the seven scores.

Questionnaire items	Score responses, n (%)						
	1	2	3	4	5	6	7
1. How much could you control events	0 (0)	4 (2.5)	10 (6.2)	27 (16.7)	59 (36.4)	50 (30.9)	12 (7.4)
2. How responsive was the VRFC^a^ to your actions	3 (1.9)	4 (2.5)	16 (9.9)	27 (16.7)	51 (31.5)	47 (29.0)	14 (8.6)
3. How natural were your interactions in the VRFC^a^	2 (1.2)	16 (9.9)	31 (19.1)	40 (24.7)	39 (24.1)	25 (15.4)	9 (5.6)
4. How completely were all your senses engaged	0 (0)	5 (3.1)	26 (16.1)	29 (17.9)	45 (27.8)	44 (27.2)	13 (8.0)
5. How much did the visual aspects of the VRFC^a^ engage you	1 (0.6)	4 (2.5)	19 (11.7)	20 (12.4)	47 (29.0)	55 (34.0)	16 (9.9)
7. How natural was the mechanism controlling movement	2 (1.2)	15 (9.3)	26 (16.1)	40 (24.7)	50 (30.9)	24 (14.8)	5 (3.1)
8. How aware were you of events in the real world around^b^	15 (9.3)	25 (15.4)	25 (15.4)	29 (17.9)	35 (21.6)	23 (14.2)	10 (6.2)
9. How aware were you of the display and controls^b^	0 (0)	3 (1.9)	19 (11.7)	30 (18.5)	53 (32.7)	37 (22.8)	20 (12.4)
10. How compelling was your sense of objects moving	0 (0)	6 (3.7)	19 (11.7)	46 (28.4)	53 (32.7)	27 (16.7)	11 (6.8)
11. How inconsistent was the information from your senses^b^	8 (4.9)	24 (14.8)	37 (22.8)	43 (26.5)	32 (19.8)	15 (8.6)	4 (2.5)
12. Consistency of experiences in VRFC^a^ with those in real food court	1 (0.6)	13 (8.0)	24 (14.8)	34 (21.0)	56 (34.6)	28 (17.3)	6 (3.7)
13. Could you anticipate happenings in response to actions	1 (0.6)	5 (3.1)	16 (9.9)	25 (15.4)	58 (35.8)	34 (21.0)	23 (14.2)
14. Completeness of searching of VRFC^a^ with your vision	0 (0)	2 (1.2)	12 (7.4)	23 (14.2)	54 (33.3)	53 (32.7)	18 (11.1)
18. How compelling was your sense of movement in VRFC^a^	2 (1.2)	7 (4.3)	23 (14.2)	29 (17.9)	50 (30.9)	34 (21.0)	17 (10.5)
19. How closely could you examine objects in the VRFC^a^	2 (0.6)	2 (1.2)	15 (9.3)	19 (12.3)	39 (24.1)	55 (34.0)	31 (18.5)
20. How well could you examine from multiple viewpoints	0 (0)	3 (1.9)	16 (9.9)	35 (21.6)	59 (36.4)	33 (19.8)	17 (10.5)
21. How well could you manipulate objects in VRFC^a^	20 (12.4)	11 (6.8)	20 (12.4)	31 (19.1)	43 (26.5)	28 (17.3)	9 (5.6)
22. Degree of confusion at end of VRFC^a^ experience^b^	24 (14.8)	33 (20.4)	31 (19.1)	16 (9.9)	38 (23.5)	16 (9.9)	4 (2.5)
23. How involved where you in the VRFC^a^	0 (0)	5 (3.1)	13 (8.0)	26 (16.1)	57 (35.2)	43 (26.5)	18 (11.1)
24. How distracting was the control mechanism^b^	12 (7.4)	26 (16.1)	41 (25.3)	37 (22.8)	29 (17.9)	12 (7.4)	5 (3.1)
25. How much was the delay between actions and outcomes^b^	51 (31.5)	47 (29.0)	25 (15.4)	19 (11.7)	13 (8.0)	6 (3.7)	1 (0.6)
26. How quickly did you adjust to the VRFC^a^	1 (0.6)	3 (1.9)	19 (11.7)	20 (12.4)	36 (22.2)	52 (32.1)	31 (19.1)
27. How proficient in movement and interaction did you feel at the end	0 (0)	4 (2.5)	11 (6.8)	41 (25.3)	50 (30.9)	38 (23.5)	18 (11.1)
28. How much did the visual display cause distraction from activities^b^	10 (6.2)	30 (18.5)	25 (15.4)	30 (18.5)	45 (27.8)	19 (11.7)	3 (1.8)
29. How much did the controls interfere with activities^b^	11 (6.8)	26 (16.1)	25 (15.4)	36 (22.2)	45 (27.8)	16 (9.9)	3 (1.9)
30. How well could you concentrate on tasks rather than the mechanisms	2 (1.2)	5 (3.1)	17 (10.5)	23 (14.2)	51 (31.5)	40 (24.7)	24 (14.8)
31. Did you learn new techniques to improve performance	6 (3.7)	13 (8.0)	14 (8.6)	24 (14.8)	54 (33.3)	36 (22.2)	15 (9.3)
32. Were you so involved in the VRFC^a^ tasks that you lost track of time^b^	7 (4.3)	11 (6.8)	24 (14.8)	34 (21.0)	41 (25.3)	31 (18.5)	15 (9.3)

^a^VRFC: virtual reality food court.

^b^Higher scores are better except for items marked with superscript a lower score is better.

**Table 3:** Total presence and presence factor scores for each factor.

Presence factor	Range^a^	Mean (SE)
Total presence	28-196	144.0 (1.4)
Control	13-91	62.1 (0.8)
Sensory	7-49	34.9 (0.4)
Realism	4-28	17.5 (0.2)
Distraction	6-42	24.0 (0.3)
Involvement	2-14	9.6 (0.2)

^a^The range indicates the minimum and maximum score possible.

## Discussion

Effective policies and regulation around food and beverages are needed to reverse the obesity epidemic [19]. There are numerous suggestions such as restricting promotions of unhealthy foods and instead promotion of healthy foods in retail outlets, energy and nutrient labeling on menu boards, increased taxes on unhealthy foods and drinks and subsidies for healthy foods [19]. Among the reasons that stakeholders may be resistant to legislation is the lack of proof of effectiveness such changes produce the desired outcomes on food consumption. Virtual reality might afford the opportunity to simulate experiments to produce evidence to enable real-world experiments. The results from the current study encourage the further development of the VRFC in order to enable a future study of food choice when conditions are manipulated in order to encourage healthier choices.

The similarities between the take-out outlets across 14 food courts simplified the process of constructing a representative VRFC. Furthermore, there were usually one or more similar chain stores such as different burger franchises or fried or barbecued chicken outlets that enabled the compilation of food and beverage products offered into a single menu for one outlet to represent these. There is considerable overlap in menus of these fast food outlets in many countries so that minimal change might be needed to use the VRFC, however, in terms of serving size and the nutritional formulation, there are noted differences for the same product between countries [20]. This would mean that country-specific nutritional databases would be needed to replace the current one developed for Australia. When the VRFC is used in future experiments to test the impact of nutritional labeling or taxation of a nutrient, such as fat or sugars, on improved diet quality, it is essential to have the appropriate nutrient database.

Participants purchased foods and drinks from every outlet indicating all were recognized and reasonable usability of the VRFC system was confirmed. Factors for control, sensory, realism, and involvement indicated the presence in the VRFC but the distraction was centered on neutral ratings. Realism measures the consistency of information from an individual’s senses and vision in the court as well as the consistency of the experience with the real world and confusion after leaving the VRFC. Distraction measures how much the mechanisms of using the VRFC interfere with the experience. However closely a virtual reality experience mimics the real world, wearing goggles and using controls are reminders this is not the real world but this does not necessarily negate the utility of virtual reality environments for food choice decisions. It has been stated that one of the most important attributes of a virtual reality environment is that the participant feels that objects in the environment are immediately actionable [21]. Our assessment demonstrated participants mostly felt in control and they experienced little delay between actions and outcomes.

Others researchers have used virtual reality to study food selection in a variety of settings and with different populations. Waterlander and colleagues have developed and validated 3-dimensional virtual reality supermarkets [12-14]. Using the UNITY platform (used in the current research) a Dutch virtual reality supermarket was designed with initial testing indicating 83% of participants found it easy to use and 79% reported their virtual purchases resembled those in the real world [14]. A recent adaptation of the software to simulate a UK supermarket was similarly tested with 83% finding it easy to use and 89% reporting it resembled their purchases in the real world [12]. The researchers provided further evidence of the validity of the virtual reality supermarket by conducting an experiment whereby they had virtual shoppers collect their till receipts at a subsequent shop at a real supermarket [13]. Seventy-four of the 123 (60.2%) completed 3 shopping experiences in this manner. The mean budget participants set for the Virtual Supermarket was NZ $121.19 (SD 65.01) but they only spent 71.4% (SD 25.6%) of their budget. Their expenditure for the four most expensive food groups (ie, fruit and vegetables, bakery goods, dairy and meat and fish in the real world) was similar to the expenditure in the virtual world.

Presence in the virtual supermarket was assessed using the Presence Questionnaire Items Stems examining the domains of sensory fidelity, focus, immersion, involvement and interface quality. Overall the participants rated their presence as medium but high scoring was noted for the interface quality [13]. One difference between the current research and the supermarket is that the experiments are conducted on a computer screen and not with headsets. One might expect that the addition of headsets would lead to greater presence. The questionnaire used in the supermarket study has some differences to that used for the VRFC but overall the ratings for factors seem comparable with moderate to high scoring.

Van Herpen et al [11] compared the effects of a real supermarket, 3-dimensional virtual supermarket and 2-dimensional photographs with an experiment on spending in 3 food categories: fruit and vegetables, milk and biscuits. As for the other supermarkets cited above, the simulation was viewed on a computer screen with keyboard and mouse navigation. Interestingly, they found similarities and differences between the real and virtual environments for the different product categories (ie, virtual reality more closely approximated the viewing), selection of products, and spending in the milk category than photographs. However, for the other 2 food categories the virtual supermarket and photographs were similar and both differed from the real supermarket. The researchers caution that while virtual reality may be useful for studying food selections and food environment interventions that the inclination to buy more foods and varieties must be accounted. However, in the current VRFC experiment subjects were only asked to purchase the food and beverage for one meal and given a fixed budget. This is a much less complex activity than completing shopping at the supermarkets which in some cases had more than 600 items on sale. It has also been reported that assigning a budget in a virtual supermarket leads to purchases closer to reality and this is why we selected an appropriate amount of money to spend, for these young adult participants who were mostly students, in the VRFC [13].

Another experiment using virtual reality environments for food choice may more closely approximate the conditions in the current study. Persky et al [15] created a virtual reality food buffet to assess how parents feed their young children. Fifty-two parents of children aged three to seven years participated in an experiment to validate the buffet by serving portions of juice and a pasta dish in the virtual and real-world settings. Both demonstrated a high correlation of virtual and real selections for the serving size. Parents also used the whole buffet to select a meal for their child and reported they were able to select a meal typical of what they might feed to their child [15].

Another use of virtual reality for food choice in differing food environments has been in the area of emotional response to foods. Gorini et al [22] have studied the differences in emotional responses in exposure to real food, virtual reality food and food photographs in patients with eating disorders. They found that the self-reported and monitored physiological responses to virtual reality food were comparable but food photographs failed to elicit the same response. Ferrer-Garcia et al [23] extended this concept to healthy subjects. They designed 4 different virtual reality scenarios to study the effects of high and low-calorie food environments in restaurant and kitchen settings on food cravings in a group of female college students. They found that the food craving elicited in the virtual environment, high-calorie food scenarios evoked stronger cravings and was the same as reported in real-world scenarios. Body mass index or self-reported subclinical eating disorder symptoms did not alter the findings. Together these two experiments validate the use of virtual reality environments to study reactions to food although not necessarily food selection in a virtual environment.

VR food environments have strengths as discussed above but it must be stated that their validity has limitations. Validity testing in two studies cited has used self-report as to whether the choices are like those in their real world [12,15]. Obviously, some reporting bias might be expected and we decided not to ask such a question in this study. It is acknowledged the Persky et al [15] study validated the serving sizes but only for one dish and beverage which may be a relatively simple exercise. The incongruent findings for different product categories in the 2 supermarket validations show we cannot be certain that when an intervention is conducted in a VR setting a positive finding can be extrapolated to the real world [11,13]. Further research on congruence between settings is required but conducting a randomized controlled trial in both virtual and real-world settings for validation simultaneously is not suggested. Rather testing in the virtual followed by the real-world is a better approach.

There are several strengths of using the VRFC over the real-world food court. Firstly, the cost of running interventions in complex real-world experiments may be prohibitive. Secondly, it removes potential conflicts of interest in collaborating with food retailers. Thirdly, we visited 14 food courts in the city and suburbs and in areas of differing socioeconomic status. As little difference in the food outlets across areas was found the virtual food court is a realistic compilation of stores and menus complete with a nutritional database of foods and prices and lastly, the participants reported an acceptable level of presence.

A limitation of the current VRFC is that we used the computer keyboard for navigation that confined them to a desk and we are unable to directly simulate sensory aspects associated with food choice such as smell and perhaps touch. Further improvements to the system are indicated and will be actioned before the VRFC is used in different experimental conditions. These include improving movement within the VRFC to make it more natural. Hand controls will be used instead of a keyboard to enable participants to move around to examine objects. Improvements in the ability to close in on the menu boards of the food outlets to examine all aspects more closely such as price and calorie labels will be enabled.

In summary, after some improvements to usability to enhance presence, the VRFC may prove useful in the conduct of experiments testing effects of taxation, pricing and promotions on food choice within this popular food environment. Obtaining such evidence is a step forward in understanding consumer behavior when changes are made to the food environment. If virtual reality studies provide positive results, the experiments must be duplicated in real-world settings to establish validity.

## Abbreviations

AUSNUT: Australian Food, Supplement and Nutrient Database
HMD: head mounted display
OECD: Organization for Economic Cooperation and Development
UK: United Kingdom
VRFC: virtual reality food court
